# Infectivity and Dissemination of Dengue Virus-1 in Different *Aedes aegypti* Populations Throughout Brazil

**DOI:** 10.3390/tropicalmed10040112

**Published:** 2025-04-19

**Authors:** Amanda Cupertino de Freitas, Ellen Santos, Lívia Baldon, Silvana de Mendonça, Fernanda Oliveira Rezende, Rafaela Moreira, Viviane Sousa, Mariana Lima, Emanuele Silva, Flávia Ferreira, João Paulo Pereira de Almeida, Siad Amadou, Bruno Marçal, Sara Comini, Marcele Rocha, Yaovi Todjro, Thiago Jiran Leite, Viviane Santos, Isaque João da Silva de Faria, Marta Giovanetti, Luiz Carlos Junior Alcantara, Luciano A. Moreira, Alvaro Ferreira

**Affiliations:** 1 Mosquitos Vetores: Endossimbiontes e Interação Patógeno-Vetor, Instituto René Rachou-Fiocruz, Belo Horizonte 30190-002, Brazil; acfreitas@aluno.fiocruz.br (A.C.d.F.); livia.baldon@aluno.fiocruz.br (L.B.); smendonca@aluno.fiocruz.br (S.d.M.); fernanda.rezende@fiocruz.br (F.O.R.); rafaela.moreira@fiocruz.br (R.M.); viviane.pauline@fiocruz.br (V.S.); alveslima.mariana@gmail.com (M.L.); bmarcal@aluno.fiocruz.br (B.M.); sara-grangeiro@live.com (S.C.); marcelebio@yahoo.com.br (M.R.); giovanetti.marta@gmail.com (M.G.); luiz.alcantara@fiocruz.br (L.C.J.A.); luciano.andrade@fiocruz.br (L.A.M.); 2 Departamento de Bioquímica e Imunologia, Instituto de Ciências Biológicas, Universidade Federal de Minas Gerais, 6627-Pampulha, Belo Horizonte 31270-901, Brazil; ellen.caroline24@gmail.com (E.S.); emanuelegsilva@gmail.com (E.S.); fvianaferreira@gmail.com (F.F.); joaopaulobio@ufmg.br (J.P.P.d.A.); gbadeguetchin@gmail.com (S.A.); todjromathias@gmail.com (Y.T.); thjfl21@gmail.com (T.J.L.); isaquejsf@gmail.com (I.J.d.S.d.F.); 3 Laboratório de Ecologia do Adoecimento & Florestas NUPEB/ICEB, Universidade Federal de Ouro Preto, Ouro Preto 35402-163, Brazil; 4 Plataforma de PCR em Tempo Real, Instituto René Rachou-Fiocruz, Belo Horizonte 30190-002, Brazil; viviane.santos@fiocruz.br; 5 Department of Sciences and Technologies for Sustainable Development and One Health, University of Campus Bio-Medico, 00128 Rome, Italy

**Keywords:** dengue virus, *Aedes aegypti*, vector competence, arbovirus, DENV-1

## Abstract

Dengue virus, one of the most prevalent mosquito-borne flaviviruses affecting humans globally, is primarily transmitted by the *Aedes aegypti* mosquito, which thrives in densely populated urban environments. Dengue incidence has surged in recent decades, becoming a major public health concern in many regions, particularly in Brazil, which has experienced recurrent outbreaks and reported over 6.6 million probable cases in the year of 2024. While the link between the mosquito vector and dengue transmission is well understood, the effects of different DENV types and their interactions with the vector capacity of natural mosquito populations are crucial for understanding disease dynamics. Here we report findings from experiments designed to analyze and compare the infectivity and dissemination of the DENV-1 strain among five *Ae. aegypti* populations collected from different regions of Brazil. When exposed to DENV-infected AG129 mice for blood feeding, these populations exhibited variations in infection rates and dissemination efficiency. Eight days post-infection, all populations demonstrated high infection rates, underscoring the substantial capacity of Brazilian *Ae. aegypti* populations to support the locally circulating DENV-1 strain. Our results demonstrate variation in *Ae. aegypti* vector competence across Brazil, revealing distinct patterns of DENV transmission efficiency. These findings highlight the necessity for geographically tailored control strategies, particularly in high-risk urban areas where outbreak potential is greatest.

## 1. Introduction

Arboviruses are viruses transmitted by arthropods to vertebrates during blood feeding [1]. Among these, the dengue virus (DENV) is a major public health concern, causing an estimated 100 to 400 million infections globally each year [2]. In recent decades, both the incidence and geographic spread of dengue have significantly increased [3], driven by factors such as climate change, rapid unplanned urbanization, and increased human mobility [4,5]. Clinically, DENV infection can manifest with high fever, headache, vomiting, and joint pain, with severe cases potentially progressing to hemorrhagic manifestations [5].

DENV, the causative agent of the disease, belongs to the *Flaviviridae* family and the *Orthoflavivirus* genus, and comprises four serotypes: DENV-1, DENV-2, DENV-3, and DENV-4 [6,7]. Its genome consists of a positive-sense RNA strand of approximately 11 kb, encoding three structural proteins, capsid (C), envelope (E), and membrane protein (prM/M), along with seven non-structural proteins (NS1, NS2A, NS2B, NS3, NS4A, NS4B, and NS5) involved in viral replication [8]. Although these non-structural proteins are functionally conserved across the four DENV serotypes, they exhibit sequence variability—particularly in NS1, NS3, and NS5—which can be used for serotype differentiation through molecular and immunological methods.

DENV is primarily transmitted by the *Aedes aegypti* mosquito during bloodmeal, when viral particles are injected into the vertebrate host along with the mosquito’s saliva [9,10]. Once inside the host, DENV can infect various cell types, including immune cells, endothelial cells, and hepatocytes [11]. Conversely, mosquitoes become infected when feeding on the blood of an infected individual. The virus initially infects the midgut and then spreads through the hemocoel to multiple tissues, eventually reaching the salivary glands, enabling transmission to another host [12]. However, not all mosquitoes become infected after feeding on a viremic host; infection depends on both the mosquito’s vector competence and the viral load present in the host at the time of feeding.

Variations in vector competence among *Aedes aegypti* populations are influenced by multiple factors, including environmental conditions, viral strain, and mosquito genetics [13,14,15,16]. In particular, genetic differences among mosquito populations can affect key aspects of viral infection, replication, and dissemination. Polymorphisms in genes involved in antiviral immunity, midgut barrier integrity, and salivary gland susceptibility have been associated with differential susceptibility to arboviruses, including DENV.

Studies indicate significant differences in vector competence for DENV among Ae. aegypti populations [17,18], largely influenced by genetic variability in both the vector and the virus [17,19,20]. In Brazil, few studies have examined variations in susceptibility and vector competence in Ae. aegypti population. However, differences in susceptibility to DENV-2 have been observed across various Brazilian cities [21]. Additionally, susceptibility to infection varies by DENV serotype, even among mosquitoes from the same locality [22].

Understanding mosquito susceptibility to viral infection across different regions is crucial for improving local arbovirus control strategies. Therefore, this study aims to evaluate the vector competence of *Ae. aegypti* from different Brazilian states for DENV-1 genotype V.

This study focuses on five Brazilian municipalities—Araraquara (SP), Jaboticatubas (MG), Petrolina (PE), Santos (SP), and Porto Alegre (RS)—which represent a diversity of biomes (Mata Atlântica, Cerrado, Caatinga, and Pampa), climates (tropical highland, semi-arid, tropical humid, and subtropical), and environmental contexts. These regions face unique challenges, such as urbanization, habitat fragmentation, waterlogged areas, and climate change, which favor the proliferation of mosquitoes and significantly increase the risk of arboviral diseases.

For instance, cities like Araraquara (São Paulo) and Jaboticatubas (Minas Gerais) have reported rising cases, with Jaboticatubas declaring a state of emergency in 2024 [23]. On the other hand, Porto Alegre (Rio Grande do Sul), which historically had low transmission, experienced an unprecedented epidemic in 2022, recording around 3897 cases [24]. Additionally, Santos (São Paulo) and Petrolina (Pernambuco) have long struggled with dengue due to environmental conditions favoring mosquito proliferation, particularly during rainy and hot seasons [25,26]. These differences in epidemiological patterns suggest potential variations in vector competence among local mosquito populations, justifying the need to investigate their susceptibility to DENV-1.

We focused on Dengue virus serotype 1 (DENV-1) due to its predominant circulation in Brazil at the time of sample collection. Historical and contemporary surveillance data indicate that DENV-1 has been responsible for several major outbreaks across the country, including in the state of São Paulo, where our study was conducted. During the 2019 epidemic, DENV-1 was the most frequently detected serotype in the Southeast region, contributing to a large proportion of reported cases. This high prevalence, combined with its recurrent involvement in outbreaks, made DENV-1 a particularly relevant target for genomic surveillance. By analyzing the genetic diversity of this serotype, we aimed to better understand the dynamics of viral dissemination and evolution in a hyperendemic setting [27].

## 2. Materials and Methods

### 2.1. Mosquito Collection

**Figure 1:**
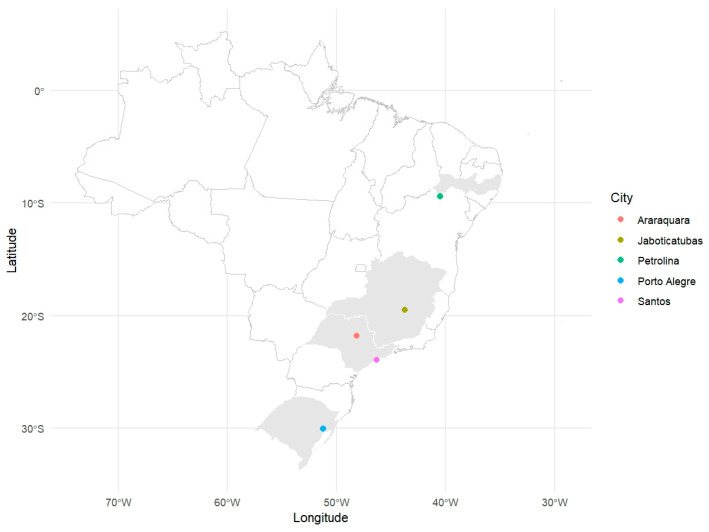
Map of Brazil showing the *Aedes aegypti* sampling sites.

Mosquito collections were conducted at five distinct locations across Brazil: Araraquara (São Paulo, ARA), Jaboticatubas (Minas Gerais, JAB), Petrolina (Pernambuco, PET), Santos (São Paulo, SAN), and Porto Alegre (Rio Grande do Sul, POA) during January and February 2022 (Figure 1). In each location, five ovitraps were distributed, and collection pallets with eggs were retrieved weekly. These locations were chosen to represent the diverse ecological and environmental conditions found throughout Brazil’s regions. The objective was to capture potential variations in mosquito populations influenced by climatic, vegetational, and anthropogenic factors. Climatic conditions at these sites included subtropical (ARA and POA), tropical highland (JAB), tropical semi-arid (PET), and tropical humid coastal (SAN) environments.

Eggs were collected using ovitraps and sent to the Mosquitos Vetores (MV) laboratory at the Instituto René Rachou-Fiocruz Minas, Belo Horizonte, Brazil. Mosquitoes were reared under insectarium-controlled conditions, maintained at 28 °C with 70–80% relative humidity and a 12/12 h light/dark cycle. The eggs were placed in plastic trays containing two liters of filtered tap water, and hatching was supported by adding 5 g of fish food (TETRA, Melle, Germany). The juvenile stages were reared at a density of 200 individuals per container. Upon adult emergence, mosquitoes were housed in 30 cm × 30 cm × 30 cm Bug Dorm insect cages and provided with 10% sucrose solution ad libitum. To establish laboratory colonies, 100 males and 100 females were used for each population (Table 1).

### 2.2. Virus Strain

To assess mosquito vector competence, we utilized a DENV-1 strain (DENV-1/H. sapiens/Brazil/Contagem/MG/BRMV09/2015), originally isolated from human blood in Contagem, Minas Gerais, Brazil, in 2015. This viral strain was propagated in C6/36 *Aedes albopictus* cells. These cells were maintained in L15 medium, enriched with 10% fetal bovine serum (FBS) and 1× Antibiotic Antimycotic (GIBCO, Invitrogen, Calrsbad, CA, USA, as previously described [28]. Cells grew to approximately 70% confluence before being infected with the virus at a multiplicity of infection (MOI) of 0.01. After the infection, cells were incubated at 28 °C for six to nine days. The viral supernatant was then harvested, clarified through centrifugation, and stored at −80 °C until use. Control supernatants (mock) were prepared using the same procedure but without virus exposure. Virus titration was conducted using plaque assays in Vero cells. The virus was allowed to adsorb onto six-well plates for 1 h at 37 °C, followed by an overlay of 2% carboxymethyl cellulose (CMC) in DMEM supplemented with 2% FBS. The plates were incubated at 37 °C with 5% CO_2_ for 5 days. Plaques were fixed with formaldehyde and stained using a solution of 70% water, 30% methanol, and 0.25% crystal violet to make them visible.

### 2.3. Mice Inoculation with DENV-1

AG129 mice were reared and kept in a specific-pathogen-free environment at the Instituto René Rachou, Fiocruz Minas, Animal Facility throughout the inoculation experiments. The mice were housed in a temperature- and humidity-controlled facility on a 12 h light/dark cycle with food and water ad libitum. We inoculated 10^6^ p.f.u. of DENV-1 into AG129 mice (IFN α/β/γ R−/−) by intraperitoneal injection (IP) in animals aged four weeks. Following inoculation, the mice were visually monitored daily and scored for morbidity and mortality. The experiments were approved by the Institutional Animal Care and Use Committee, Comissão de Ética no Uso de Animais da Fiocruz (CEUA) and performed according to institutional guidelines (license number LW-26-20).

### 2.4. Mosquito Infection with DENV-1

Thirty female mosquitoes (maximum per cage, 5–7 days old) were placed in each mesh-covered container (0.88 mm nylon) and divided into 4- and 8-day post-infection (d.p.f) groups. Following 24 h sugar deprivation, blood feeding was conducted using viremic AG129 mice anesthetized with ketamine/xylazine (80/8 mg kg^−1^) at 2 d.p.f. Feeding rates varied between 50 and 100% across experimental replicates, reflecting natural variation in mosquito feeding behavior. Only blood-engorged females were retained for subsequent infection analyses. Then, anesthetized and unshaved mice were positioned in a prone orientation on top of the netting-covered containers containing female mosquitoes, allowing the entire ventral surface and limbs to be accessible to the mosquitoes. A maximum of thirty female mosquitoes were allowed to feed on one mouse for 30 min. All five mosquito populations were fed on the same DENV-1-infected mouse. The viremia of the mouse was measured at the time of blood feeding, and the viral titers were consistently above 10⁶ PFU/mL. To ensure a natural route of virus acquisition and enhance feeding efficiency, mosquitoes were allowed to feed directly on viremic AG129 mice, rather than through artificial membrane feeding, as this approach better mimics and resembles the natural process of blood meal acquisition from a viremic host. After blood feeding, females that were fully engorged were selected. These mosquitoes were placed in a container covered with nylon mesh with a cotton pad soaked with 10% glucose solution and with a plastic cup with soaked paper on the bottom for egg laying. Afterward, mosquitoes were harvested individually for tissue dissection and subsequent RNA extraction at four- and eight-days post-feeding. To assess the DENV-1 infection rates, we calculated the proportion of infected midguts relative to the total number of mosquitoes that were fed blood from each population. To assess the dissemination rate of DENV-1 infection in the mosquitoes, we calculated the proportion of infected carcasses relative to the total number of mosquitoes that were fed blood from each population.

### 2.5. RNA Extraction and RT-qPCR

RNA extraction from mosquito samples was conducted using the TRIzol reagent method (Invitrogen, Carlsbad, CA, USA), with some modifications to the manufacturer’s protocol [29]. Briefly, whole mosquitoes were individually placed in a 1.5 mL Eppendorf tube, followed by the addition of 200 µL of TRIzol and two glass beads. The samples were then vigorously ground in a bead beater for 90 s. Afterward, they were incubated at room temperature for 10 min. Next, 40 µL of chloroform was added, and the mixture was agitated vigorously for 30 s. After another 10-min incubation at room temperature, the samples were centrifuged at 12,000× *g* for 15 min at 4 °C.

The supernatant was combined with an equal volume of chilled isopropanol and mixed gently for 2 min and then left to precipitate overnight at −20 °C. Following this, the samples were centrifuged again at 12,000× *g* for 5 min at 4 °C. The resulting pellet was washed with 75% (*v*/*v*) ethanol and air-dried for approximately 10 min. Finally, the purified RNA was dissolved in 10 µL of RNase-free water and stored at −80 °C [28].

The extracted total RNA was reverse transcribed using M-MLV reverse transcriptase (Promega, Madison, WI, USA) along with random primers for initiation. Negative controls included both non-infected mosquitoes and reactions omitting reverse transcriptase. Positive controls utilized serial DENV-1 RNA dilutions (1:1 to 1:10,000) in each run to validate primer efficiency. All real-time PCR experiments were performed on the QuantStudio 12K Real-Time PCR System (Applied Biosystems, Foster City, CA, USA), utilizing the SYBR Green PCR Master Mix (Applied Biosystems—Life Technologies, Foster City, CA, USA) for the amplification process. The final reaction volume was set at 10 µL [28].

The thermal cycling conditions included a Hold Stage (fast ramp to 95 °C and hold for 20 s); a PCR Stage (40 cycles of 95 °C, hold for 15 s, fast ramp to 60 °C, and hold for 60 s); and a Melt Stage (fast ramp to 95 °C, hold for 15 s, fast ramp to 60 °C, hold for 1 min, and slow ramp of 0.05 °C/s to 95 °C, and then a hold for 15 s). All real-time PCR reactions were performed in triplicate. The relative quantification of gene expression was assessed using the 2^−ΔCt^ method, as previously described [30,31,32]. In this approach, the Ct (cycle threshold) values for the target gene (DENV-1) were normalized against the Ct values of the internal reference gene (housekeeping gene RPL32) within the same sample. Viral load was estimated by quantitative RT-PCR using the ΔCt method, where the Ct value of the DENV target gene was normalized to the Ct value of the *Aedes aegypti* housekeeping gene RPL32 (ΔCt = Ct_DENV-Ct_RPL32); results were expressed as relative viral RNA levels using the 2^−ΔCt^ formula and plotted on a log10 scale [28]. This normalization approach was applied without an external reference sample, as the study focused on comparing viral load within individual samples or across experimental conditions where a baseline reference was neither applicable nor required. The viral RNA load was expressed in relation to the endogenous control housekeeping gene, RPL32, for both *Ae. aegypti* and AG129 mice. For *Ae. aegypti*, the *RPL32* primers were as follows: (Forward) 5′-AGC CGC GTG TTG TAC TCT G-3′ and (Reverse) 5′-ACTTCT TCG TCC GCT TCT TG-3′. For mice, the *RPL32* primers were as follows: (Forward) 5′-GCTGCC ATC TGT TTT ACG G-3′ and (Reverse) 5′-TGA CTG GTG CCT GAT GAA CT-3′. For DENV-1, the primers were as follows: (Forward) 5′-TCG GAA GCT TGC TTA ACG TAG-3′ and (Reverse) 5′-TCC GTT GGT TGT TCA TCA GA-3′ [28].

### 2.6. Statistical Analyses

Statistical evaluations were conducted with R software (version 4.4.1; www.r-project.org, accessed on 14 June 2024). To explore the relationship between mosquito populations (predictor variable) and infection status (binary outcome), we utilized logistic regression within a Generalized Linear Model (GLM), specifying the family argument as “binomial” and the link function as “logit,” and applied likelihood-ratio χ^2^ tests. For comparing viral loads in midgut and carcass samples across different mosquito populations, we conducted a Kruskal–Wallis test, followed by Dunn’s test for pairwise comparisons. Statistical significance was defined as *p*-values less than 0.05 [33]. For the statistical analysis of viral loads, only samples in which viral RNA was detected by RT-qPCR were included; samples with undetectable viral RNA were excluded from mean viral load calculations and comparative analyses, which were performed using the non-parametric Kruskal–Wallis test.

## 3. Results

### 3.1. Differential Susceptibility of Brazilian Aedes aegypti Strains to DENV-1 Infection

**Figure 2:**
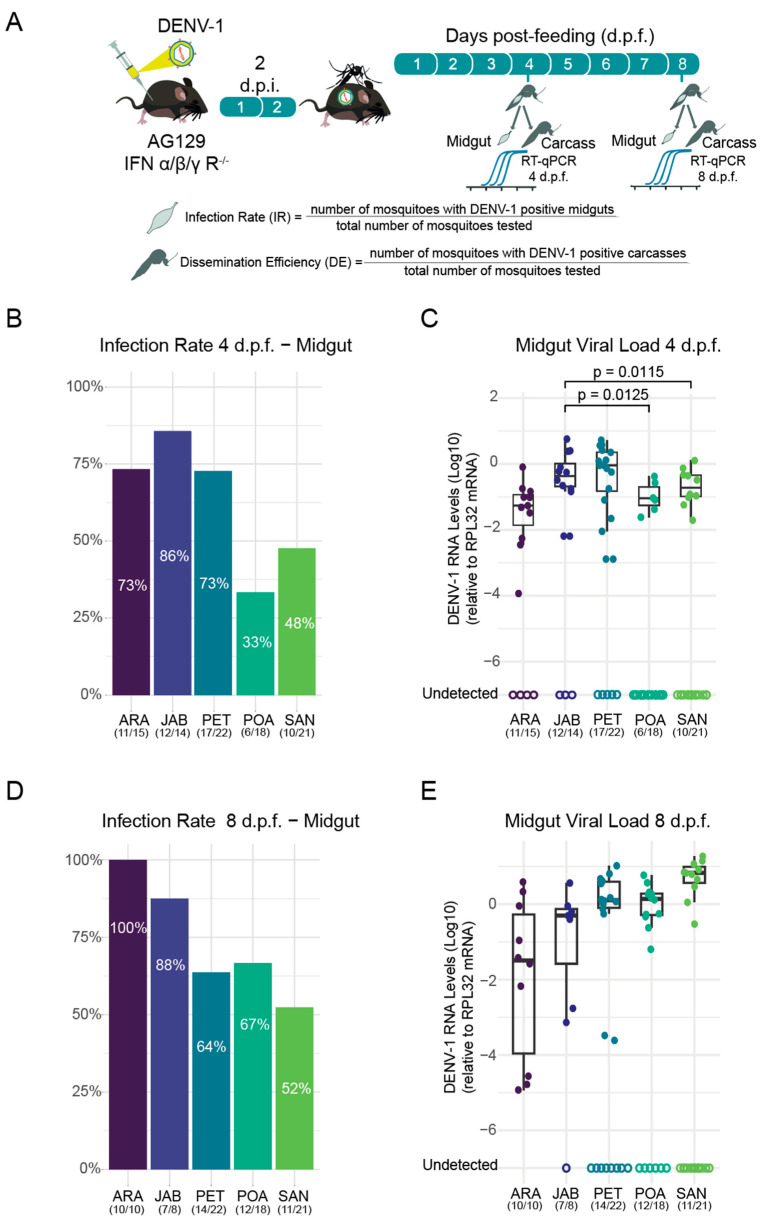
Infection rates of DENV-1 across Brazilian *Aedes aegypti* populations. (**A**) Scheme of experimental design used. Infection rates at 4 (**B**) and 8 (**D**) days post-feeding (d.p.f.) are shown. DENV-1 RNA levels of each mosquito tested are shown, and significant differences (**C**,**E**) are indicated by the *p*-value (Kruskal–Wallis test). The number of mosquitoes with detected DENV-1 RNA out of the total number of mosquitoes tested is presented below the population abbreviation. To quantify the DENV-1 load, we utilized the 2^−ΔCt^ (delta Ct) method. The mosquitoes used to obtain the results in both the midgut and the carcass were the same individuals from the cohort. Empty circles represent samples where viral RNA was not detected. Samples with undetectable viral RNA were excluded from mean viral load calculations and comparative analyses.

Once the five populations were established (Figure 1) in the laboratory, three-week-old AG129 mice were infected with DENV-1 via intraperitoneal injections. After three days, the mice were anesthetized and exposed to female mosquitoes from the five populations for blood feeding to assess the mosquitoes’ susceptibility to DENV-1 infection (Figure 2A). Mosquitoes were collected at four- and eight-day post-feeding (d.p.f.), followed by dissection. The midgut and other tissues, collectively referred to as the carcass, were carefully removed for further analysis. After dissection, the midgut of each mosquito was subjected to RNA extraction, followed by RT-qPCR to assess infection rates in the populations. The infection rate was calculated as the proportion of midguts infected with DENV-1 relative to the total number of mosquitoes that had fed in each population. Our results demonstrate that all populations were susceptible to DENV-1 infection as soon as 4 days post-feeding, though significant differences were observed between populations (*p*-value) (Figure 2B). At 4 d.p.f., the JAB population exhibited the highest midgut infection rate (85.71%), followed by ARA (73.33%) and PET (72.73%). The populations with the lowest infection rates were SAN (47.62%) and POA (33.33%).

In addition to assessing infection rates, RT-qPCR analysis of midgut tissue provided quantification of DENV-1 viral load across the five mosquito populations. Significant differences in viral load were detected at 4 days post-feeding (d.p.f.), with the JAB population exhibiting substantially higher viral loads than both POA (*p* = 0.012) and SAN (*p* = 0.0114; Kruskal–Wallis test; Figure 2C).

By 8 d.p.f., infection rates in the midgut had increased across all populations, though with notable variation in infection intensity. The ARA population exhibited the highest susceptibility, reaching 100% midgut infection, whereas other populations showed lower rates. Among the analysed populations, JAB showed the highest infection rate (87.5%), followed by POA (66.7%), PET (63.6%), and SAN (52.4%) (Figure 2D). However, it should be noted that JAB had a smaller sample size than the other populations. The markedly lower infection rate in SAN, nearly half that of ARA, highlights substantial differences in susceptibility among these populations. Despite these variations in infection prevalence, the mean viral loads in midguts at 8 d.p.f. did not differ significantly between the tested populations (Figure 2E).

### 3.2. Variation in DENV-1 Dissemination Rates Among Ae. aegypti Populations

**Figure 3:**
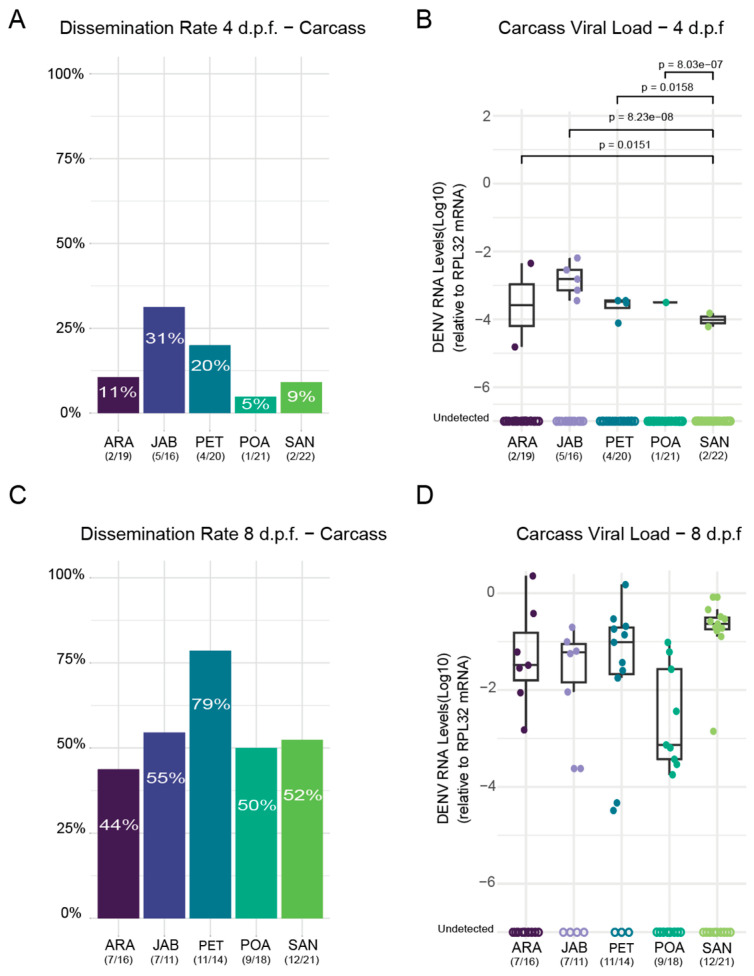
Dissemination efficiencies of DENV-1 at 4 (**A**) and 8 (**C**) days post-feeding (d.p.f.) are shown. The number of mosquitoes tested is indicated below the population abbreviation. The DENV-1 RNA levels of each mosquito tested at 4 (**B**) and 8 (**D**) days post-feeding (d.p.f.) are shown, and significant differences are indicated by the *p*-value (Kruskal–Wallis test). The number of mosquitoes with detected DENV-1 RNA out of the total number of mosquitoes tested is presented below the population abbreviation. Empty circles represent samples where viral RNA was not detected. To quantify the DENV-1 load, we utilized the 2^−ΔCt^ (delta Ct) method. The mosquitoes used to obtain the results in both the midgut and the carcass were the same individuals from the cohort. Samples with undetectable viral RNA were excluded from mean viral load calculations and comparative analyses.

The results revealed that viral dissemination already occurred in all populations at 4 d.p.f., although the rates varied and did not exceed 32% (Figure 3A).

The JAB population exhibited the highest dissemination rate at 31.2%, followed by PET at 20%. The remaining populations showed lower dissemination rates, with ARA at 10%, SAN at 9%, and POA at 5%. To complement the assessment of dissemination rates, RT-qPCR analysis quantified DENV-1 viral load in midgut tissues across the five mosquito populations, revealing significant variations among them (Figure 3B).

At 8 d.p.f., viral dissemination increased across all mosquito populations. The PET population exhibited the highest dissemination rate of DENV-1 at 78.6%, followed by JAB (55%), SAN (52%), and POA (50%) (Figure 3C). In contrast, the ARA population had the lowest dissemination rate, at 43.8%. Despite this increase in dissemination, no significant differences in DENV-1 viral load were observed among the populations in midgut tissues at 8 d.p.f. (Figure 3D).

## 4. Discussion

Dengue is one of the most widespread arboviral diseases globally, with an estimated 390 million infections annually [29,33]. In Brazil, dengue is endemic [34], with the first major epidemic recorded in Boa Vista, Roraima, between 1981 and 1982, caused by DENV-1 and DENV-4 [23]. Since then, all four serotypes have been detected across the country [35,36]. Despite prevention efforts, including vector control, mosquito nets, repellents, and vaccines, Latin America remains the epidemic’s epicenter. In 2024, Brazil experienced an unprecedented dengue outbreak, reporting approximately 6.6 million probable cases and 6,230 deaths—an alarming 300% increase from 2023 [37]. This drastic surge underscores the need to investigate the factors driving this increase, particularly the vector competence of Brazilian *Ae. aegypti* populations, to better understand their role in dengue transmission and inform more effective control strategies.

To assess the susceptibility of *Ae. aegypti* populations from different Brazilian states to DENV-1, we conducted experiments evaluating the infectivity and dissemination of a DENV-1 strain across five mosquito populations from distinct regions. When exposed to DENV-infected AG129 mice for blood feeding, most populations exhibited high infection rates, with some reaching up to 100%; however, infection rates varied between populations. Additionally, our findings indicate that Brazilian *Ae. aegypti* populations are highly susceptible to viral dissemination from the midgut to other tissues, as evidenced by DENV-1 detection in the carcass (mosquito body excluding the midgut), with dissemination efficiency exceeding 50%. To evaluate infection rates, we analysed dengue RNA presence in the midgut, the first organ infected after a blood meal [38]. At 4 d.p.f, all populations were susceptible to DENV-1 infection, with Jaboticatubas and Araraquara exhibiting the highest infection rates. By 8 d.p.f, all midguts from Araraquara were infected, indicating a 100% infection rate. The Jaboticatubas population appeared to partially control midgut to some extent, though this may be due to the limited number of midguts analysed. At 4 d.p.f, Jaboticatubas and Araraquara showed midgut infection rates of 86% and 73%, respectively, while at 8 d.p.f, the rates were 88% and 100%.

At 4 d.p.f, Jaboticatubas exhibited the highest DENV-1 dissemination rate (31.2%), consistent with its high midgut infection susceptibility. Viral dissemination requires overcoming the mosquito’s primary immune barrier—the midgut [39]. Consequently, dissemination rates are typically lower than midgut infection rates, especially at early time points (e.g., 4 d.p.f). However, by 8 d.p.f, the Petrolina population displayed the highest dissemination rate, followed by Jaboticatubas and Araraquara. These findings align with previous CHIKV infection studies [39], where these same populations exhibited the highest dissemination rates (91% for all three populations at 8 d.p.f.). By 8 d.p.f, all populations demonstrated high infection rates, highlighting the significant vector competence of Brazilian *Ae. aegypti* for the locally circulating DENV-1 strain. These results correlate with epidemiological data from the analysed cities.

Araraquara, São Paulo, is an endemic dengue region [40]. In 2023, the city reported 797 probable dengue cases, rising to 1769 by early October 2024 [37]. Prior studies have linked dengue incidence in Araraquara to precipitation, temperature, and humidity fluctuations [37]. Additionally, DENV-1, DENV-2, and DENV-3 have been detected in the region over different years [41,42].

Conversely, Jaboticatubas, Minas Gerais, reported 409 probable dengue cases in 2023, escalating to 1196 in 2024 [37], prompting a state of emergency [43]. While limited research exists on arboviruses in Jaboticatubas, DENV-2 has been documented [44]. The high susceptibility and dissemination capacity of DENV-1 in the local *Ae. aegypti* population, coupled with the highest viral load among the analysed populations, raise concerns about future outbreaks.

In contrast, Petrolina, Pernambuco, has historically exhibited lower dengue incidence. A study from 2015 and 2017 reported the lowest dengue incidence among analysed cities in Pernambuco, likely due to low precipitation levels [45]. However, local *Ae. aegypti* populations have shown high susceptibility to DENV-2, with elevated infection rates detected in the midgut, salivary glands, and fat body [46]. Our findings reinforce the need for enhanced surveillance, as high dissemination rates may indicate an increased risk of local transmission. Notably, while the Petrolina population demonstrated high dissemination, this does not necessarily confirm active transmission, as salivary glands and saliva were not separately analysed, leaving some uncertainty regarding transmission potential.

Cities with lower infection and dissemination rates, such as Porto Alegre, Rio Grande do Sul, have historically reported low dengue incidence [47]. The first autochthonous dengue case was recorded in 2010 [48], with most cases in the city being imported, largely due to human travel between 2013 and 2016 [49]. The low dengue occurrence in Porto Alegre may be linked to reduced *Ae. aegypti* susceptibility to DENV-1, despite the serotype’s continued presence in the region since 2010 [47,50].

Santos, São Paulo, has experienced a sharp rise in dengue cases since the virus was first recorded there in 1997 [51]. In 2023, the city reported 717 probable cases, surging to 5275 by early October 2024 [41], highlighting the growing public health burden. Surprisingly, however, our results suggest that the local *Ae. aegypti* population exhibits low susceptibility to DENV-1, despite documented circulation of this serotype [51,52]. This discrepancy suggests that other mosquito species may contribute to dengue transmission. Supporting this hypothesis, previous studies have detected DENV-3 in *Ae. albopictus* larvae and DENV-1 in adult *Ae. aegypti* females [52].

Genetic variations, such as polymorphisms in immune-related genes, can affect viral susceptibility, replication efficiency, and overall mosquito immune responses. Additionally, environmental conditions experienced during development—such as microbiota composition—may influence adult mosquito physiology and vector competence. Although these factors were not directly investigated in this study, they may contribute to the observed phenotypic differences and should be explored in future research to better understand the mechanisms underlying vector competence.

In this study, we used RT-qPCR to assess viral load in mosquitoes. While this method offers high sensitivity and reliable quantification of viral RNA, it is important to acknowledge that factors such as RNA degradation, variability in sample handling, and differences in RNA extraction efficiency may introduce some degree of variation in the resulting viral load estimates.

## 5. Conclusions

Overall, our findings underscore significant variation in *Ae. aegypti* vector competence across Brazilian regions, emphasizing the need for region-specific dengue control strategies. Given the dramatic rise in dengue cases in 2024, understanding mosquito susceptibility and viral dissemination is critical for predicting transmission patterns and implementing effective public health interventions. Further studies are needed to explore the genetic, environmental, and immunological factors contributing to regional differences in *Ae. aegypti* competence for DENV-1.

## Figures and Tables

**Table 1:** *Aedes aegypti* populations collected in Brazil.

Name	City	State	Species	Stage	Number of Mosquitoes	Generation	Year
ARA	Araraquara	São Paulo	*Aedes aegypti*	eggs	100 females + 100 males	F2	2022
JAB	Jaboticatubas	Minas Gerais	*Aedes aegypti*	eggs	100 females + 100 males	F2	2022
PET	Petrolina	Pernambuco	*Aedes aegypti*	eggs	100 females + 100 males	F2	2022
POA	Porto Alegre	Rio Grande do Sul	*Aedes aegypti*	eggs	100 females + 100 males	F2	2022
SAN	Santos	São Paulo	*Aedes aegypti*	eggs	100 females + 100 males	F2	2022

## Data Availability

The data presented in this study are openly available in FigShare. https://doi.org/10.6084/m9.figshare.28509563 (accessed on 27 February 2025).

## References

[1] Figueiredo L.T.M. (2007). Emergent Arboviruses in Brazil. Rev. Soc. Bras. Med. Trop..

[2] World Health Organization (2024). Dengue and Severe Dengue.

[3] World Health Organization (2024). Dengue—Global Situation.

[4] Kraemer M.U., Sinka M.E., Duda K.A., Mylne A.Q., Shearer F.M., Barker C.M., Moore C.G., Carvalho R.G., Coelho G.E., Van Bortel W. (2015). The Global Distribution of the Arbovirus Vectors *Aedes aegypti* and *Ae. Albopictus*. eLife.

[5] Kok B.H., Lim H.T., Lim C.P., Lai N.S., Leow C.Y., Leow C.H. (2023). Dengue Virus Infection—A Review of Pathogenesis, Vaccines, Diagnosis and Therapy. Virus Res..

[6] Postler T.S., Beer M., Blitvich B.J., Bukh J., de Lamballerie X., Drexler J.F., Imrie A., Kapoor A., Karganova G.G., Lemey P. (2023). Renaming of the Genus Flavivirus to Orthoflavivirus and Extension of Binomial Species Names within the Family Flaviviridae. Arch. Virol..

[7] Zanotto P.M., Gould E.A., Gao G.F., Harvey P.H., Holmes E.C. (1996). Population Dynamics of Flaviviruses Revealed by Molecular Phylogenies. Proc. Natl. Acad. Sci. USA.

[8] Perera R., Kuhn R.J. (2008). Structural Proteomics of Dengue Virus. Curr. Opin. Microbiol..

[9] Lambrechts L., Scott T.W., Gubler D.J. (2010). Consequences of the Expanding Global Distribution of Aedes Albopictus for Dengue Virus Transmission. PLoS Negl. Trop. Dis..

[10] Weng S.-C., Tsao P.-N., Shiao S.-H. (2021). Blood Glucose Promotes Dengue Virus Infection in the Mosquito *Aedes aegypti*. Parasites Vectors.

[11] Martina B.E.E., Koraka P., Osterhaus A.D.M.E. (2009). Dengue Virus Pathogenesis: An Integrated View. Clin. Microbiol. Rev..

[12] Raquin V., Lambrechts L. (2017). Dengue Virus Replicates and Accumulates in *Aedes aegypti* Salivary Glands. Virology.

[13] Lambrechts L., Paaijmans K.P., Fansiri T., Carrington L.B., Kramer L.D., Thomas M.B., Scott T.W. (2011). Impact of Daily Temperature Fluctuations on Dengue Virus Transmission by *Aedes aegypti*. Proc. Natl. Acad. Sci. USA.

[14] Yan J., Kim C.-H., Chesser L., Ramirez J.L., Stone C.M. (2023). Nutritional Stress Compromises Mosquito Fitness and Antiviral Immunity, While Enhancing Dengue Virus Infection Susceptibility. Commun. Biol..

[15] Gaye A., Wang E., Vasilakis N., Guzman H., Diallo D., Talla C., Ba Y., Dia I., Weaver S.C., Diallo M. (2019). Potential for Sylvatic and Urban *Aedes* Mosquitoes from Senegal to Transmit the New Emerging Dengue Serotypes 1, 3 and 4 in West Africa. PLoS Negl. Trop. Dis..

[16] Fansiri T., Fontaine A., Diancourt L., Caro V., Thaisomboonsuk B., Richardson J.H., Jarman R.G., Ponlawat A., Lambrechts L. (2013). Genetic Mapping of Specific Interactions between *Aedes aegypti* Mosquitoes and Dengue Viruses. PLoS Genet..

[17] Black W.C., Bennett K.E., Gorrochótegui-Escalante N., Barillas-Mury C.V., Fernández-Salas I., de Lourdes Muñoz M., Farfán-Alé J.A., Olson K.E., Beaty B.J. (2002). Flavivirus Susceptibility in *Aedes aegypti*. Arch. Med. Res..

[18] Failloux A.-B., Vazeille M., Rodhain F. (2002). Geographic Genetic Variation in Populations of the Dengue Virus Vector *Aedes aegypti*. J. Mol. Evol..

[19] Lequime S., Fontaine A., Ar Gouilh M., Moltini-Conclois I., Lambrechts L. (2016). Genetic Drift, Purifying Selection and Vector Genotype Shape Dengue Virus Intra-Host Genetic Diversity in Mosquitoes. PLoS Genet..

[20] Salazar M.I., Richardson J.H., Sánchez-Vargas I., Olson K.E., Beaty B.J. (2007). Dengue Virus Type 2: Replication and Tropisms in Orally Infected *Aedes aegypti* Mosquitoes. BMC Microbiol..

[21] Lourenço-de-Oliveira R., Vazeille M., De Filippis A.M.B., Failloux A.B. (2004). *Aedes aegypti* in Brazil: Genetically Differentiated Populations with High Susceptibility to Dengue and Yellow Fever Viruses. Trans. R. Soc. Trop. Med. Hyg..

[22] Chaves B.A., Godoy R.S.M., Campolina T.B., Júnior A.B.V., Paz A.d.C., Vaz E.B.d.C., Silva B.M., Nascimento R.M., Guerra M.d.G.V.B., Lacerda M.V.G. (2022). Dengue Infection Susceptibility of Five *Aedes aegypti* Populations from Manaus (Brazil) after Challenge with Virus Serotypes 1–4. Viruses.

[23] Nogueira R.M.R., Araújo J.M.G., Schatzmayr H.G. (2007). Dengue Viruses in Brazil, 1986–2006. Rev. Panam. Salud Publica.

[24] Breda R., Motta A.S. (2024). Análise da influência de determinantes meteorológicos na periodicidade de epidemias de dengue em Porto Alegre. Ver. Inst. Adolfo Lutz. São Paulo.

[25] dos Santos S.D., Ribeiro M.C.S.A. (2021). Incidence of Dengue and Socioeconomic and Entomological Indicators in Santos, São Paulo, 2012–2016. Nurs. Ed. Bras..

[26] De Freitas J.R., Santiago E.J.P., De Freitas J.C.R., Da Silva A.S.A., De Araújo Filho R.N., Piscoya V.C., Cunha Filho M. (2020). Space-temporal analysis trend of the numbers of dengue cases in Pernambuco-Brazil. Res. Soc. Dev..

[27] Bezerra J.M.T., de Sousa S.C., Tauil P.L., Carneiro M., Barbosa D.S. (2021). Entry of dengue virus serotypes and their geographic distribution in Brazilian federative units: A systematic review. Rev. Bras. Epidemiol..

[28] Baldon L.V.R., de Mendonça S.F., Ferreira F.V., Rezende F.O., Amadou S.C.G., Leite T.H.J.F., Rocha M.N., Marques J.T., Moreira L.A., Ferreira A.G.A. (2022). AG129 Mice as a Comprehensive Model for the Experimental Assessment of Mosquito Vector Competence for Arboviruses. Pathogens.

[29] World Health Organization (2009). Dengue: Guidelines for Diagnosis, Treatment, Prevention and Control.

[30] Schmittgen T.D., Livak K.J. (2008). Analyzing real-time PCR data by the comparative C(T) method. Nat. Protoc..

[31] Livak K.J., Schmittgen T.D. (2001). Analysis of relative gene expression data using real-time quantitative PCR and the 2(-Delta Delta C(T)) Method. Methods.

[32] Olmo R.P., Ferreira A.G.A., Izidoro-Toledo T.C., Aguiar E.R.G.R., de Faria I.J.S., de Souza K.P.R., Osório K.P., Kuhn L., Hammann P., de Andrade E.G. (2018). Control of dengue virus in the midgut of Aedes aegypti by ectopic expression of the dsRNA-binding protein Loqs2. Nat. Microbiol..

[33] Bhatt S., Gething P.W., Brady O.J., Messina J.P., Farlow A.W., Moyes C.L., Drake J.M., Brownstein J.S., Hoen A.G., Sankoh O. (2013). The Global Distribution and Burden of Dengue. Nature.

[34] Araújo V.E.M.D., Bezerra J.M.T., Amâncio F.F., Passos V.M.D.A., Carneiro M. (2017). Aumento Da Carga de Dengue No Brasil e Unidades Federadas, 2000 e 2015: Análise Do Global Burden of Disease Study 2015. Rev. Bras. Epidemiol..

[35] Villabona-Arenas C.J., De Oliveira J.L., Capra C.D.S., Balarini K., Loureiro M., Fonseca C.R.T.P., Passos S.D., Zanotto P.M.D.A. (2014). Detection Of Four Dengue Serotypes Suggests Rise In Hyperendemicity In Urban Centers Of Brazil. PLoS Negl. Trop. Dis..

[36] Bastos M.D.S., Figueiredo R.M.P.D., Ramasawmy R., Itapirema E., Gimaque J.B.L., Santos L.O., Figueiredo L.T.M., Mourão M.P.G. (2012). Simultaneous Circulation of All Four Dengue Serotypes in Manaus, State of Amazonas, Brazil in 2011. Rev. Soc. Bras. Med. Trop..

[37] Gurgel-Gonçalves R., Oliveira W.K., Croda J. (2024). The Greatest Dengue Epidemic in Brazil: Surveillance, Prevention, and Control. Rev. Soc. Bras. Med. Trop..

[38] Carpenter A., Clem R.J. (2023). Factors Affecting Arbovirus Midgut Escape in Mosquitoes. Pathogens.

[39] De Freitas A., Rezende F., De Mendonça S., Baldon L., Silva E., Ferreira F., Almeida J., Amadou S., Marçal B., Comini S. (2024). The High Capacity of Brazilian *Aedes aegypti* Populations to Transmit a Locally Circulating Lineage of Chikungunya Virus. Viruses.

[40] Luna E.J.A., Figueiredo G.M., Levi J.E., Campos S.R.S.L.C., Felix A.C., Souza N.S.E., Figueiredo W.M., Costa A.A., Cardoso M.R.A., Pannuti C.S. (2020). A Cohort Study to Assess the Incidence of Dengue, Brazil, 2014–2018. Acta Trop..

[41] Ferreira A.C., Chiaravalloti Neto F., Mondini A. (2018). Dengue in Araraquara, State of São Paulo: Epidemiology, Climate and *Aedes aegypti* Infestation. Rev. Saúde Pública.

[42] De Souza C.S., Caleiro G.S., Claro I.M., De Jesus J.G., Coletti T.M., Da Silva C.A.M., Costa Â.A., Inenami M., Ribeiro A.C., Felix A.C. (2024). Phylogenetics, Epidemiology and Temporal Patterns of Dengue Virus in Araraquara, São Paulo State. Viruses.

[43] Prefeitura de Jaboticatubas (2024). Prefeitura Decreta Situação de Emergência Devido ao Aumento de Casos de Dengue no Município. https://www.jaboticatubas.mg.gov.br/index.php/mais-noticias/602-prefeitura-decreta-situa%C3%A7%C3%A3o-de-emerg%C3%AAncia-devido-ao-aumento-de-casos-de-dengue-no-munic%C3%ADpio.html.

[44] Adelino T.É.R., Giovanetti M., Fonseca V., Xavier J., De Abreu Á.S., Do Nascimento V.A., Demarchi L.H.F., Oliveira M.A.A., Da Silva V.L., De Mello A.L.E.S. (2021). Field and Classroom Initiatives for Portable Sequence-Based Monitoring of Dengue Virus in Brazil. Nat. Commun..

[45] Do Nascimento I.D.S., Pastor A.F., Lopes T.R.R., Farias P.C.S., Gonçales J.P., Do Carmo R.F., Durães-Carvalho R., Da Silva C.S., Silva Júnior J.V.J. (2020). Retrospective Cross-Sectional Observational Study on the Epidemiological Profile of Dengue Cases in Pernambuco State, Brazil, between 2015 and 2017. BMC Public Health.

[46] Carvalho-Leandro D., Ayres C.F.J., Guedes D.R.D., Suesdek L., Melo-Santos M.A.V., Oliveira C.F., Cordeiro M.T., Regis L.N., Marques E.T., Gil L.H. (2012). Immune Transcript Variations among *Aedes aegypti* Populations with Distinct Susceptibility to Dengue Virus Serotype 2. Acta Trop..

[47] Gregianini T.S., Tumioto-Giannini G.L., Favreto C., Plentz L.C., Ikuta N., Da Veiga A.B.G. (2018). Dengue in Rio Grande Do Sul, Brazil: 2014 to 2016. Rev. Med. Virol..

[48] Da Cruz Ferreira D.A., Degener C.M., De Almeida Marques-Toledo C., Bendati M.M., Fetzer L.O., Teixeira C.P., Eiras Á.E. (2017). Meteorological Variables and Mosquito Monitoring Are Good Predictors for Infestation Trends of *Aedes aegypti*, the Vector of Dengue, Chikungunya and Zika. Parasites Vectors.

[49] Guzzetta G., Marques-Toledo C.A., Rosà R., Teixeira M., Merler S. (2018). Quantifying the Spatial Spread of Dengue in a Non-Endemic Brazilian Metropolis via Transmission Chain Reconstruction. Nat. Commun..

[50] Tumioto G.L., Gregianini T.S., Dambros B.P., Cestari B.C., Alves Nunes Z.M., Veiga A.B.G. (2014). Laboratory Surveillance of Dengue in Rio Grande Do Sul, Brazil, from 2007 to 2013. PLoS ONE.

[51] Bellegarde Fernandes M.A., Natal D., Domingos M.D.F. (2014). Aspectos Epidemiológicos Da Transmissão De Dengue Em Santos, São Paulo, No Período De 1997 A 2012. J. Health Biol. Sci..

[52] de Figueiredo M.L., Gomes A.d.C., Amarilla A.A., Leandro A.d.S., Orrico A.d.S., de Araujo R.F. (2010). Mosquitoes Infected with Dengue Viruses in Brazil. Virol. J..

